# 

**Published:** 1893-02

**Authors:** 


### Postponement.

The Annual Meeting of the Mississippi Valley Dental Society, which is usually held in the first week of March, has been postponed for one year. The President, after consulting with the Executive Committee and a number of the more prominent members of the Society, has decided that it will be better to lay over this meeting for one year. So many other important meetings are near at hand, and rapidly approaching, that attention is largely occupied in their behalf, and it is well nigh a certainty that should an attempt be made to hold this annual meeting, very few would be in attendance, and the Executive Committee have had very little encouragement in the way of preparation of papers for the meeting, and so upon the whole it has been thought best that the meeting be omitted for this year with the hope that by March 1894 renewed energy will have been gathered, and an old-time meeting of this body will be held.

By the direction of the President, Dr. O. N. Heise.

### THE DENTAL REGISTER,

A MONTHLY MAGAZINE DEVOTED TO THE INTERESTS OF THE

**DENTAL PROFESSION.**

*Terms Reduced to* $2.00 *Per Year. Single Copies,* 25 *Cents.*

**EDITED BY PROF. J. TAFT, D.D.S.**

—AND—

**Published by SAM'L A. CROCKER & CO., Ohio Dental and Surgical Depot.**

The volume commences in January and closes in December.

The REGISTER being issued monthly is always fresh, therefore Indispensable to the practitioner who desires to keep posted up with the new inventions and improvements which are daily developed. Neither expense nor effort will be spared to give value and variety to its contents. Articles will be illustrated with well executed engravings whenever they will add to the interest or assist to explanation.

Its extensive circulation and frequency of issue make it one of the BEST DENTAL ADVERTISING MEDIUMS in the United States.

All subscriptions must begin with January or July.

Local or special notices, five lines or less, will be inserted for $3 each insertion; over five lines, 50 cts. per line.

All advertising bills payable in advance, or at furthest, after the issue of the first number containing the advertisement. All transient advertisements to be paid for in advance.

☞CONTRIBUTIONS SOLICITED.

Exclusively Editorial Communications should be addressed to the Editor.

The latest number of the REGISTER may be ordered with or without other goods at any time, and all letters should be addressed to

**SAM'L A. CROCKER & CO., Ohio Dental and Surgical Depot, Cincinnati, O.**

